# TGFβ1 Signalling in human mesenchymal stem cells is regulated by the primary cilium

**DOI:** 10.1186/2046-2530-4-S1-P20

**Published:** 2015-07-13

**Authors:** M Labour, S Christensen, D Hoey

**Affiliations:** 1 Centre for Applied Biomedical Engineering Research, University of Limerick, Casteltroy, Ireland; 2 Department of Mechanical Aeronautical and Biomedical Engineering, University of Limerick, Casteltroy, Ireland; 3 Materials and Surface Science Institute, University of Limerick, Casteltroy, Ireland; 4 Department of Biology, University of Copenhagen, Copenhagen, Denmark; 5 Trinity Centre for Bioengineering, Trinity College, Dublin, Ireland

## Objective

Mesenchymal Stem Cells (MSCs) are mobilized in response to injury to initiate healing and remodelling. TGFβ1 is known to induce MSC migration and homing in various tissues including bone. However, the molecular mechanisms involved are poorly understood. TGFβ1 signalling has recently been linked to the primary cilium in fibroblasts. This cellular microdomain, enriched in transmembrane receptors, could be a specialized centre for TGFβ signalling. Therefore, the aim of this study is to investigate the role of the primary cilium in TGFβ1-induced MSC migration.

## **Methods**

TGFβ1 induced MSC migration was investigated using a Boyden chamber approach. The localization of TGFβ1 signalling components within MSCs was investigated through immunocytochemistry. Deletion of the primary cilia was performed with siRNA targeting Ift88.

## Results

Firstly, the constitutively active TGFβRII is expressed in human MSCs and localizes preferentially to primary cilia in up to 80% of ciliated cells. TGFβRI, recruited upon ligand binding to TGFβRII is also localized to the primary cilia (14% of ciliated cells), and this specific localization increases after TGFβ1 stimulation (33% of ciliated cells). This result suggests a recruitment of the receptor into the ciliary microdomain. Moreover, the downstream signalling phosphorylated Smad2 and Smad4 localized to primary cilia. Furthermore, TGFβ1 induces human and mouse MSCs chemotaxis and interestingly, after deletion of the primary cilia, the migration capacity is decreased.

## Conclusions

These results reveal that TGFβ1 signalling cascade occurs within the microdomain of the primary cilium and more importantly the primary cilium is involved in TGFβ1-induced MSC migration.

